# Functional development of midbrain and pons during human gestation and electrophysiological markers for DS intervention

**DOI:** 10.1016/j.jare.2025.10.046

**Published:** 2025-10-30

**Authors:** Yongkang Wu, Yingmei Fu, Hao Peng, Yanni Chen, Fanglin Wang, Jian Wang, Jinli Ou, Yigang Dong, Qingmiao Hu, Yan Cui, Yuhua Zheng, Jianqiang Huang, Yi Dong, Qizhi He

**Affiliations:** a Department of Pathology, Shanghai Key Laboratory of Maternal Fetal Medicine, Shanghai Institute of Maternal Fetal Medicine and Gynecologic Oncology, Shanghai First Maternity and Infant Hospital, School of Medicine, Tongji University, Shanghai 200092, China; b Key Laboratory of Adolescent Health Assessment and Exercise Intervention of Ministry of Education, East China Normal University, Shanghai 200241, China; c Shanghai Key Laboratory of Psychotic Disorders, Brain Health Institute, National Center for Mental Disorders, Shanghai Mental Health Center, Shanghai Jiao Tong University School of Medicine, Shanghai 200030, China; d Clinical and Translational Research Center, Shanghai First Maternity and Infant Hospital, School of Medicine, Tongji University, Shanghai 200092, China; e Department of Stem Cell and Regenerative Medicine, Institute of Pathology and Southwest Cancer Center, Southwest Hospital, Chongqing 400038, China; f Computer Network Information Center, Chinese Academy of Sciences, Beijing 100083, China; g Hangzhou Institute for Advanced Study, University of Chinese Academy of Sciences, Hangzhou 310024, China; h University of Chinese Academy of Sciences, Beijing 100049, China

## Abstract

The early fetal period (GW8-20) is crucial for establishing motor and cognitive neural circuits. However, ethical constraints and limitations in current research methods have hindered our understanding of functional development in related brain regions during this period. This study presents the first functional map of midbrain-pons development in human fetuses from GW10 to GW17, systematically investigating ion channel development, the dynamic evolution of excitatory and inhibitory networks, and synaptic maturation, thereby addressing a critical developmental research gap. Employing electrophysiological techniques and Smart-seq technology, we revealed the spatiotemporal dynamics of neuronal excitability, synaptic transmission, and synaptic plasticity in the human midbrain-pons, highlighting functional developmental differences between healthy fetuses and those with Down syndrome (DS) at GW17. Our results demonstrate that the midbrain-pons complex undergoes a rapid phase of neural network maturation from GW10 to GW17, characterized by a swift increase in neuronal excitability, gradual maturation of synaptic transmission, and enhanced synaptic plasticity. In addition, during mid-gestation, the midbrain and pons follow distinct developmental trajectories, exhibiting regional specificity and non-linear maturation patterns. The midbrain-pons functional map addresses a significant gap in developmental neuroscience and challenges existing organoid models by incorporating region-specific network features into disease modeling. In DS fetuses at GW17, we observed early functional deficits, including decreased action potential firing frequency, unbalanced AMPAR/NMDAR-mediated EPSC ratio and sEPSCs/sIPSCs ratio, impaired synaptic plasticity. Electrophysiological profiling reveals that by gestational week 17, fetuses with Down syndrome already manifest signs of neural network dysfunction. By correlating early electrophysiological markers with neurodevelopmental pathology, we provide new potential markers for future prenatal diagnosis of DS and suggest a critical intervention window (GW13.5-17).

## Highlights

• First Functional Map of Midbrain-Pons Development: The study presents the first functional map of midbrain and pons development in human fetuses from GW10 to GW17, revealing changes in neuronal excitability, synaptic transmission, and plasticity during this critical developmental window.
• Functional Maturation of Midbrain-Pons Networks: The midbrain–pons complex undergoes non-linear developmental progression from GW10 to GW17, displaying regional asynchrony between structural and functional maturation. After GW13.5, the midbrain advances synaptic network maturation, whereas the pons predominantly develops morphology and fiber projections.
• Early Functional Deficits in Down Syndrome (DS) Fetuses: GW17 DS fetuses show reduced neuronal excitability, disrupted excitatory–inhibitory balance, and impaired synaptic plasticity, revealing candidate biomarkers and a critical window for early intervention.

## Introduction

The formation of functional neural circuits during early fetal development (8–20 weeks gestation) provides the basis for postnatal brain function. During this critical period, a cascade of regulation—such as ion channel dynamics, synaptic pruning, and critical periods of plasticity—facilitates the transition of neural circuits from structural to functional states. The midbrain-pons complex acts as a dual hub for motor regulation and Cognition**.** Dopaminergic neurons in the substantia nigra modulate the balance between initiation and inhibition in the basal ganglia-thalamocortical loop through D1/D2 receptor heterogeneity. Meanwhile, glutamatergic relay neurons in the pons facilitate real-time sensorimotor integration in corticospinal and cerebellar pathways via frequency encoding [[1], [2], [3]]. The maturation of these circuits establishes the embryological foundation for advanced functions, such as motor coordination and spatial cognition. However, ethical barriers to obtaining fetal tissue, along with limitations of traditional functional analysis techniques, have left the early functional development map of the human brainstem and its pathological patterns unresolved [4].

Current research on human fetal brain development primarily focuses on the later stages of pregnancy (GW20 and beyond) [5,6], with an emphasis on postnatal neurodevelopment and behavioral phenotypes. However, the early developmental period (GW8-20), crucial for establishing subcortical motor and sensory integration circuits, remains underexplored at the cellular, molecular, and network levels [7]. Ethical limitations on the acquisition of fetal tissue have led existing research to focus primarily on morphological changes [8,9] (e.g., neurogenesis gradients and axonal guidance gradients), with limited exploration of the maturation of functional circuits [10]. Traditional structural MRI can track macroscopic developmental processes (e.g., cortical folding and white matter tract formation) but cannot resolve the finer details of neuronal electrical activity or functional synaptic connections [11]. Although high-throughput genomics can identify gene variants and potential genetic risks in early development [[12], [13], [14], [15]]. Related studies reveal a close association between genetic variations in genes such as FGFR2, DYRK1A, DNMT3L, and TBX1 with intellectual disabilities and motor impairments [[16], [17], [18]]. But these methods do not elucidate how molecular changes correlate with neural dysfunction and behavioral changes. For instance, it remains unclear whether ion channel changes detected at the transcript level in human fetuses (e.g., the transition from Nav1.3 to Nav1.6) synchronize with the maturation of functional sodium currents [19,20]. Stem cell-derived brain organoids, while simulating early functional development, exhibit physiological characteristics and network complexity that differ significantly from those of the human brain (e.g., γ oscillation power in organoids is only about 30 % of that in fetal brain tissue at the same stage) [[21], [22], [23]]. Moreover, they lack the capacity to model regions like the pons nuclei [24], preventing the reconstruction of topological connections in midbrain- pons pathways (e.g., dopaminergic projections from the substantia nigra to the striatum) [[25], [26], [27]]. These multi-scale limitations hinder the construction of a functional developmental map of the human brain during early pregnancy and prevent full replication of its complexity and cellular diversity.

In vertebrates, during the assembly of central motor circuits, the midbrain- pons complex creates the developmental blueprint for motor coordination and sensory integration through a spatiotemporally specific functional topology [28]. Dopaminergic neurons in the substantia nigra pars compacta dynamically encode both the initiation and inhibition of movement, maintaining motor control balance [29,30]. The pons nuclei achieve time compression and spatial encoding of sensory-motor information through cross-frequency coupling, particularly θ-γ oscillations [31]. While these functional properties have been extensively studied in adult neural networks, how this complex hierarchical regulatory network is established during embryonic development remains unclear [32]. Specifically, it remains unclear whether the midbrain- pons hub adheres to the synchronization principle across molecular, cellular, and functional levels during early development. Does the development of the midbrain- pons complex during early fetal stages follow a synchronization principle across molecular and electrophysiological scales? Down syndrome (DS), a genetic disorder resulting from trisomy of chromosome 21, is associated with motor deficits, intellectual impairments, and other abnormalities [33,34]. Recent advances in high-throughput omics technologies have gradually uncovered the molecular regulatory networks underlying DS-associated neural circuits. For instance, DYRK1A disrupts the radial migration of midbrain dopaminergic neurons and impairs Netrin-1-dependent axonal guidance mechanisms in the brainstem nuclei, consequently affecting the topological assembly of motor pathways. However, the dynamic regulation of neural functional activity by molecular and structural abnormalities during early development remains unclear. Furthermore, due to the complexity of the human brain, brain models cannot provide a systematic explanation of the pathological features [35,36].

In responsive to these challenges, this study systematically analyzes electrophysiology and gene expression of midbrain-pons glutamate neurons in human fetal midbrain-pons region. In addition, we constructs a spatiotemporal map of its functional development during the period from GW10 to GW17. Our investigation focuses on: (1) dynamic evolution of excitatory activity; (2) development of AMPA/NMDA receptor dynamics; (3) temporal differences in the excitatory/inhibitory network balance; and (4) temporal window dependence of long-term potentiation (LTP). By comparing GW17 DS fetuses with healthy controls, we identify functional developmental abnormalities in DS embryos, providing a solid theoretical basis for developing prenatal diagnostic biomarkers and intervention strategies for the critical window period. In conclusion, this study constructs a systematic map of early fetal brain functional development and lays the groundwork for the early diagnosis and precise intervention of neurodevelopmental disorders.

## Materials and methods

**1. Human sample collection**

Human fetal tissue samples were obtained from patients undergoing elective pregnancy termination at the Pingdingshan Maternity and Infant Health Hospital.

**2. Patch-clamp recordings**

Fetal midbrain and pons slices were transferred to the electrophysiology room, where the recording solution was kept at 28 ± 2 °C. Neurons for whole-cell recording were selected using a 40× Nikon microscope, based on smooth morphology, well-defined contours, three-dimensional appearance, and the presence of clear synaptic structures. The recording solution consisted of artificial cerebrospinal fluid (ACSF) containing 124 mM NaCl, 4.4 mM KCl, 2 mM CaCl_2_, 1 mM MgSO_4_, 2 mM NaHCO_3_, 1 mM NaH_2_PO_4_, and 10 mM glucose, saturated with oxygen and carbon dioxide to maintain pH balance. The electrode impedance ranged from 4 to 7 MΩ, and only cells with Ra < 20 MΩ were included in the analysis following seal formation.

### Action potential recording from midbrain and pons neurons

Action potentials were recorded in current-clamp mode using a potassium gluconate electrode solution (120 mM K-gluconate, 6 mM HEPES, 10 mM EGTA, 0.1 mM Mg-ATP, 4 mM Na-GTP, pH 7.30, osmolarity 280 mOsm/kg). Tetrodotoxin (TTX) was applied to block Na+ channels. A current injection of −50 pA was applied for 500 ms, followed by 10-step increments of 10 pA to assess action potential firing.

### Recording AMPAR/NMDAR mediated EPSCs from midbrain and pons neurons

EPSCs mediated by AMPAR and NMDAR were recorded in voltage-clamp mode using a CsCl electrode solution (120 mM CsCl, 10 mM HEPES, 1 mM EGTA, 2 mM Mg-ATP, 0.3 mM Na-GTP, 5 mM QX-314, pH 7.25, osmolarity 285 mOsm/kg). The stimulating electrode was positioned in the presynaptic fiber bundle to evoke 50 % maximal EPSC with a 0.1 Hz single-pulse stimulation. AMPAR currents were recorded at −70 mV (to relieve Mg^2+^ blockade of NMDAR), with 50 μM AP-5 added to block NMDAR. NMDAR currents were recorded at  0 mV, with 20 μM CNQX added to block AMPARs, and the ACSF was replaced with a Mg^2+^-free solution..Each neuron was stimulated three times, and the average of the values was used for analysis.

### Recording spontaneous excitatory and inhibitory currents from midbrain and pons neurons

Spontaneous excitatory postsynaptic currents (sEPSCs) were recorded in voltage-clamp mode using a potassium gluconate electrode solution, with the holding potential set to −70 mV (near the AMPAR reversal potential). Bicuculline (10 μM) was added to block GABA receptors, and data were recorded for 5 min. Signals with a rise time < 2 ms and a decay time constant of 5–15 ms were included in the analysis. For spontaneous inhibitory postsynaptic currents (sIPSCs), a CsCl electrode solution was used with the holding potential set to 0 mV to enhance Cl^−^ current resolution. 20 μM CNQX and 50 μM AP-5 were added to block AMPAR and NMDAR, and data were recorded for 10 min with the criteria for inclusion: rise time < 1 ms, decay time constant 10–30 ms.

### Recording EPSC-LTP from midbrain and pons neurons

LTP of EPSCs was induced and recorded using a low-chloride electrode solution (110 mM CsMeSO_3_, 10 mM HEPES, 10 mM EGTA, 5 mM QX-314, 4 mM Mg-ATP, 0.3 mM Na-GTP, 5 mM phosphocreatine, pH 7.25, osmolarity 285 mOsm/kg). QX-314 was used to block postsynaptic sodium channels, and phosphocreatine was included to maintain intracellular energy levels. The stimulating electrode (impedance 2–4 MΩ) was placed in the presynaptic fiber bundle. Baseline recordings were made with 0.1 Hz single-pulse stimulation (50 % maximal EPSC intensity) for 10 min. LTP was induced by theta-burst stimulation (100 Hz for 1 s, repeated three times at 20-second intervals), followed by 0.1 Hz stimulation and continuous EPSC recording for 30 min. LTP was considered successful if the EPSC amplitude increased by >20 %. The data collection criteria were as follows: EPSC rise time < 3 ms and decay time constant >10 ms to ensure synaptic specificity.

**3. Smart-seq**

Following completion of patch-clamp recordings, target neurons were gently aspirated with a glass microelectrode under controlled negative pressure. All procedures were performed in RNase-free PBS pre-cooled to 4 °C, and the aspiration volume was restricted to less than 5 μL to minimize contamination from neighboring cells.

**4. Data analysis**

Image processing was performed with Adobe Photoshop and ImageJ. Electrophysiological data were analyzed using Clampfit 10 and MiniAnalysis. Statistical analyses were conducted with Origin 2022 and GraphPad Prism 10, employing Kruskal–Wallis, Mann–Whitney U, and Student’s t tests as appropriate. RNA-seq data were processed and generated through the cloud platform of Shanghai Meiji Biomedical Technology Co., Ltd. All values are reported as mean ± SEM.

## Result

**Fig. 1:**
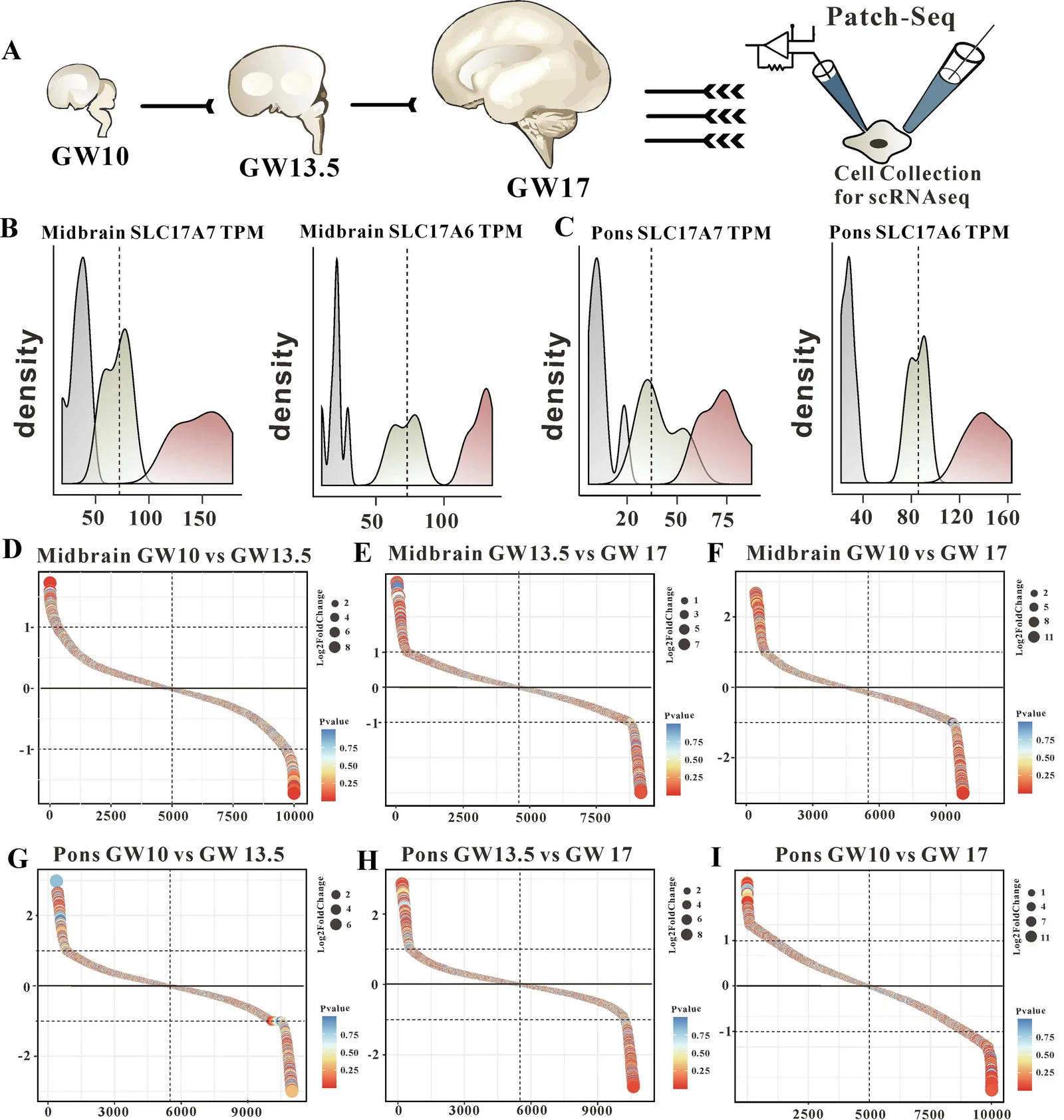
**Sample collection and data recording at termination of pregnancy gestational development** (A) Samples were collected at GW10, GW13.5, and GW17, and cells from the midbrain and pons were subjected to electrophysiological recordings and Smart-seq analysis. (B-C) mRNA expression of SLC17A7 and SLC17A6 in midbrain and pons glutamatergic neurons ,gray represents GW10, green represents GW13.5, and red represents GW17. (D) Gene Ranking Dotplot of midbrain glutamatergic neurons at GW10 and GW13.5. (E) Gene Ranking Dotplot of midbrain glutamatergic neurons at GW135 and GW17. (F) Gene Ranking Dotplot of midbrain glutamatergic neurons at GW10and GW17. (G) Gene Ranking Dotplot of pons glutamatergic neurons at GW10 and GW13.5. (H) Gene Ranking Dotplot of pons glutamatergic neurons at GW13.5 and GW17. (I) Gene Ranking Dotplot of pons glutamatergic neurons at GW10 and GW17.

Electrophysiological techniques were used to record action potential (AP), AMPAR/NMDAR mediated currents, spontaneous excitatory currents (sEPSCs), spontaneous inhibitory currents (sIPSCs), and EPSC-LTP in motor-related brain regions (midbrain, pons) of human fetuses at 10, 13.5, and 17 weeks of gestation (Fig. 1A). Recorded neurons underwent Smart-seq sequencing, and those expressing glutamatergic markers SLA171A and SLA171 were selected for functional assays and RNA analysis (Fig. 1B/C).

**1. Time-dependent dynamic changes in neuronal excitability and membrane conductance in the midbrain and pons**

**Fig. 2:**
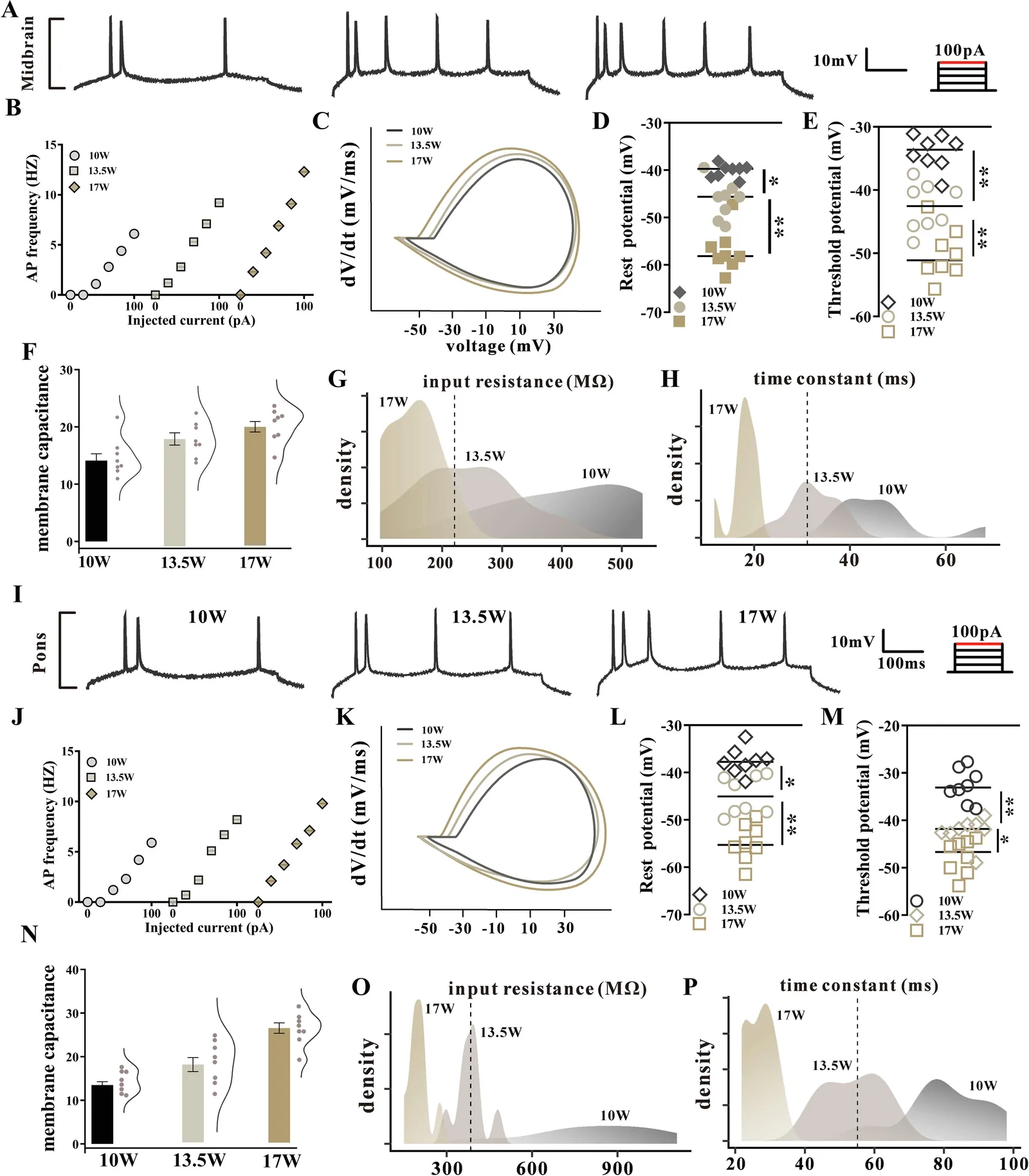
**Electrophysiological properties of midbrain and pons neurons during gestational development** (A) Action potential (AP) Schematic of midbrain neurons at GW10, GW13.5 and GW17. (B) AP firing frequency of midbrain neurons versus injected current. (C) First derivative of AP (dV/dt) in midbrain neurons. (D) Resting membrane potential (RMP) of midbrain neurons. (E) AP threshold potential in midbrain neurons. (F) Membrane capacitance of midbrain neurons. (G) Input resistance of midbrain neurons. (H) Membrane time constant of midbrain neurons. (I) AP of pons neurons at GW10, GW13.5 and GW17. (J) AP firing frequency of pons neurons versus injected current. (K) First derivative of APs (dV/dt) in pons neurons. (L) RMP of pons neurons. (M) AP threshold potential in pons neurons. (N) Membrane capacitance of pons neurons. (O) Input resistance of pons neurons. (P) Membrane time constant of pons neurons. Data from 8 neurons per region. Data are presented as mean ± SEM. *p < 0.01, **p < 0.05.

Electrophysiological recordings reveal significant time-dependent changes in neuronal excitability and membrane conductance in the human fetal motor-related brain regions (midbrain and pons) during early development (GW10-GW17). In the midbrain, as fetal age increased, the maximum firing frequency of AP significantly increased (Fig. 2A; GW10: 2.4 Hz vs GW17: 5.8 Hz, p < 0.05). This trend is consistent with results from human cortical organoid studies [22], non-human primate motor cortex [37] development, and the growth pattern of action potential frequency observed in human midbrain organoids cultured in vitro for 60–90 days (equivalent to gestational weeks 14–18) [38]. Notably, compared to GW10, the resting membrane potential (RMP) of neurons at GW17 became more hyperpolarized (Fig. 2D; GW10: −40.19 mV vs GW17: −57.02 mV, p < 0.01), indicating the gradual maturation of voltage-gated potassium channels. This suggests that the temporal expression of voltage-gated potassium channels is a conserved mechanism of excitability regulation across species [39], though differences remain compared to adult dopaminergic neurons in the midbrain [40]. Additionally, the AP threshold shifted significantly in a negative direction from GW10 to GW17 (Fig. 1E; GW10: −34.08 mV vs GW17: −50.11 mV, p < 0.01), increasing neuronal sensitivity to synaptic input and laying the physiological foundation for high-frequency oscillations in later-stage neural networks [41]. This trend aligns with the observed maturation pattern of motor networks in human brain studies. The developmental features of membrane conductance parameters further illustrate the dynamic remodeling of neuronal function over time. We found that membrane capacitance (Cm) of midbrain neurons significantly increased between 10 and 13.5 weeks (Fig. 2F; GW10: 14.10 pF vs GW13.5: 18.74 pF, p < 0.01), indicating rapid increase in the area of dendrite/axonal membranes. However, between 13.5 and 17 weeks, the growth rate of Cm slowed (Fig. 2F; GW13.5: 18.74 pF vs GW17: 20.75 pF, p＞0.05). This shift may mark the onset of myelination [42], transitioning from membrane expansion toward enhanced conduction efficiency and indicating a more mature state of neural network function. This developmental trajectory coincides with the time window (GW13-GW15) when spontaneous fetal limb movements first appear [43,44], indicating that the maturation of midbrain glutamatergic neurons may drive the generation of early motor patterns. Moreover, compared to GW10, the input resistance of midbrain neurons significantly decreased at GW17, and the time constant also decreased synchronously (Fig. 2G/H). This change suggests that during development from GW10 to GW17, the density of voltage-gated ion channels in neurons increased, membrane conductance enhanced, and the speed of neural network information integration improved. This indicates a transition from a rapid growth phase in early development to a phase of quality optimization, in which electrophysiological adjustments enable neurons to meet the demands of complex information processing and faster signal transmission. In the pons, although changes in AP threshold, time constant, and input resistance showed trends similar to those in the midbrain, the growth pattern of Cm differed (Fig. 2N; GW10: 13.48 pF vs GW13.5: 17.89 pF, p < 0.05; GW13.5: 17.89 pF vs GW17: 26.30 pF, p < 0.01). In contrast to midbrain neurons, pons glutamatergic neurons continued to increase membrane capacitance, indicating preferential development of descending fibers in the motor conduction pathway. This developmental pattern is reported here for the first time in the human fetal nervous system [[6], [45]].

**2. Spatiotemporal developmental patterns of AMPAR/NMDAR mediated synaptic transmission**

**Fig. 3:**
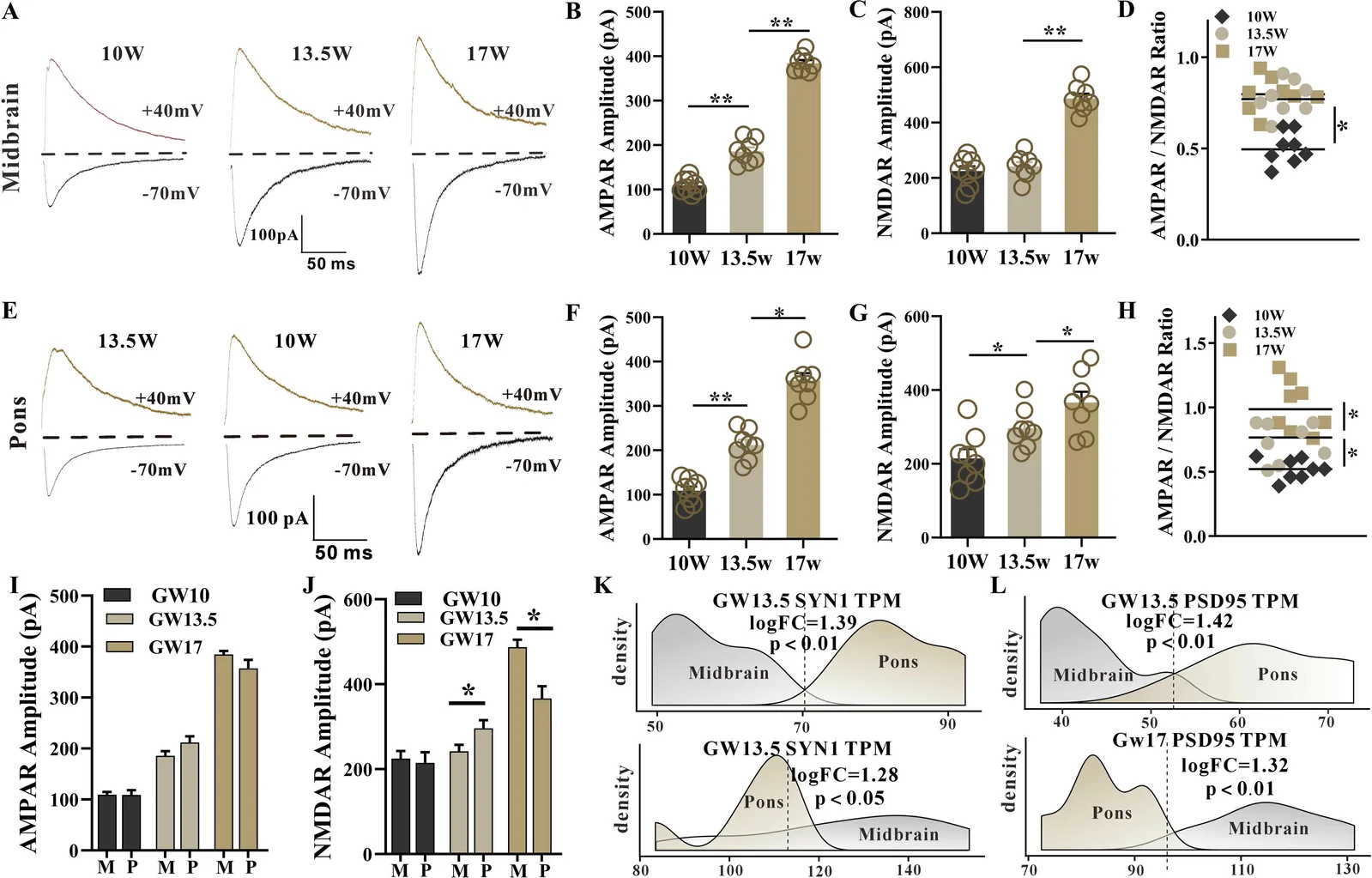
**AMPAR/NMDAR-mediated synaptic currents in midbrain and pons neurons** (A) Schematic of AMPAR/NMDAR-mediated EPSCs in midbrain neurons at GW10, GW13.5, and GW17. (B) AMPAR-EPSCs amplitude in midbrain neurons. (C) NMDAR-EPSCs amplitude in midbrain neurons. (D) AMPAR/NMDAR currents ratio in midbrain neurons. (E) Schematic of AMPAR/NMDAR-mediated EPSCs in pons neurons at GW10, GW13.5, and GW17. (F) AMPAR-EPSCs amplitude in pons neurons. (G) NMDAR-EPSCs amplitude in pons neurons. (H) AMPAR/NMDAR current ratio in pons neurons. (I) Comparison of AMPAR-EPSCs in glutamatergic neurons across developmental time points. (J) Comparison of and NMDAR-EPSCs in glutamatergic neurons across developmental time points. (K) Differences in SYN1 mRNA expression between midbrain and pons glutamatergic neurons at GW13.5 and GW17. (L) Differences in PSD95 mRNA expression between midbrain and pons glutamatergic neurons at GW13.5 and GW17. Data from 8 neurons per region. Data are presented as mean ± SEM. *p < 0.01, **p < 0.05.

Electrophysiological recordings reveal stage-specific maturation characteristics of glutamatergic synaptic transmission mediated by AMPAR and NMDAR in human fetal motor-related brain regions during GW10–17. In the midbrain, the amplitude of AMPAR mediated EPSCs increases stepwise with gestational age: compared to GW10, the current significantly increases at GW13.5 (Fig. 3B; GW10: 109.36 pA vs GW13.5: 185.54 pA, p < 0.01), and further increases at GW17 (Fig. 3B; GW13.5: 185.54 pA vs GW17: 384.58 pA, p < 0.01), suggesting upregulation of AMPAR expression. In contrast, NMDAR-EPSCs remain stable from GW10 to GW13.5 (Fig. 3C; GW10: 224.76 pA vs GW13.5: 241.96 pA, p＞0.05), but show a delayed increase from GW13.5 to GW17 (Fig. 3C; GW13.5: 241.97 pA vs GW17: 487.322 pA, p < 0.01). NMDAR-EPSCs remained stable between GW10 and GW13.5, potentially reflecting the subunit switch from NR2B to NR2A, consistent with transcriptomic findings in macaques [46]. Further analysis of dynamic changes in the AMPAR/NMDAR-EPSCs ratio revealed that at GW10, the AMPAR/NMDAR-EPSCs ratio was 0.50 (Fig. 3D), indicating that NMDAR dominated and AMPAR expression was minimal [47]. At GW 13.5, the ratio increased to 0.77 (Fig. 3D), suggesting that AMPAR were inserted in large amounts into the postsynaptic membrane. Similar developmental patterns were observed in the pons, where AMPAR-EPSCs at GW13.5 and GW17 were significantly enhanced compared to GW10 (Fig. 3F). The increasement of NMDAR-EPSCs in the pons occurred earlier than in the midbrain, showing significant growth between GW10 and GW13.5 (Fig. 3G; GW10: 214.91 pA vs GW13.5: 296.08 pA, p＜0.05), and further increased between GW13.5 and GW17 (Fig. 3G; GW13.5: 276.58 pA vs GW17: 366.44 pA, p＜0.01). From GW10 to GW17, NMDAR-mediated EPSCs displayed distinct temporal dynamics. At GW13.5, NMDAR-EPSC amplitudes were greater in pons glutamatergic neurons than in midbrain neurons, whereas by GW17 they were higher in midbrain neurons than in pons neurons (Fig. 3I/J). Consistently, SYN1 and PSD95 mRNA expression followed a similar temporal trajectory (Fig. 3K/L). These findings indicate that from GW10.5 to GW13.5, excitatory synapse maturation in the midbrain lags behind that in the pons. However, between GW13.5 and GW17, midbrain excitatory synaptic development accelerates relative to the pons.

**3. Synergistic enhancement of excitatory and inhibitory synapses and the development of LTP in the midbrain and pons**

**Fig. 4:**
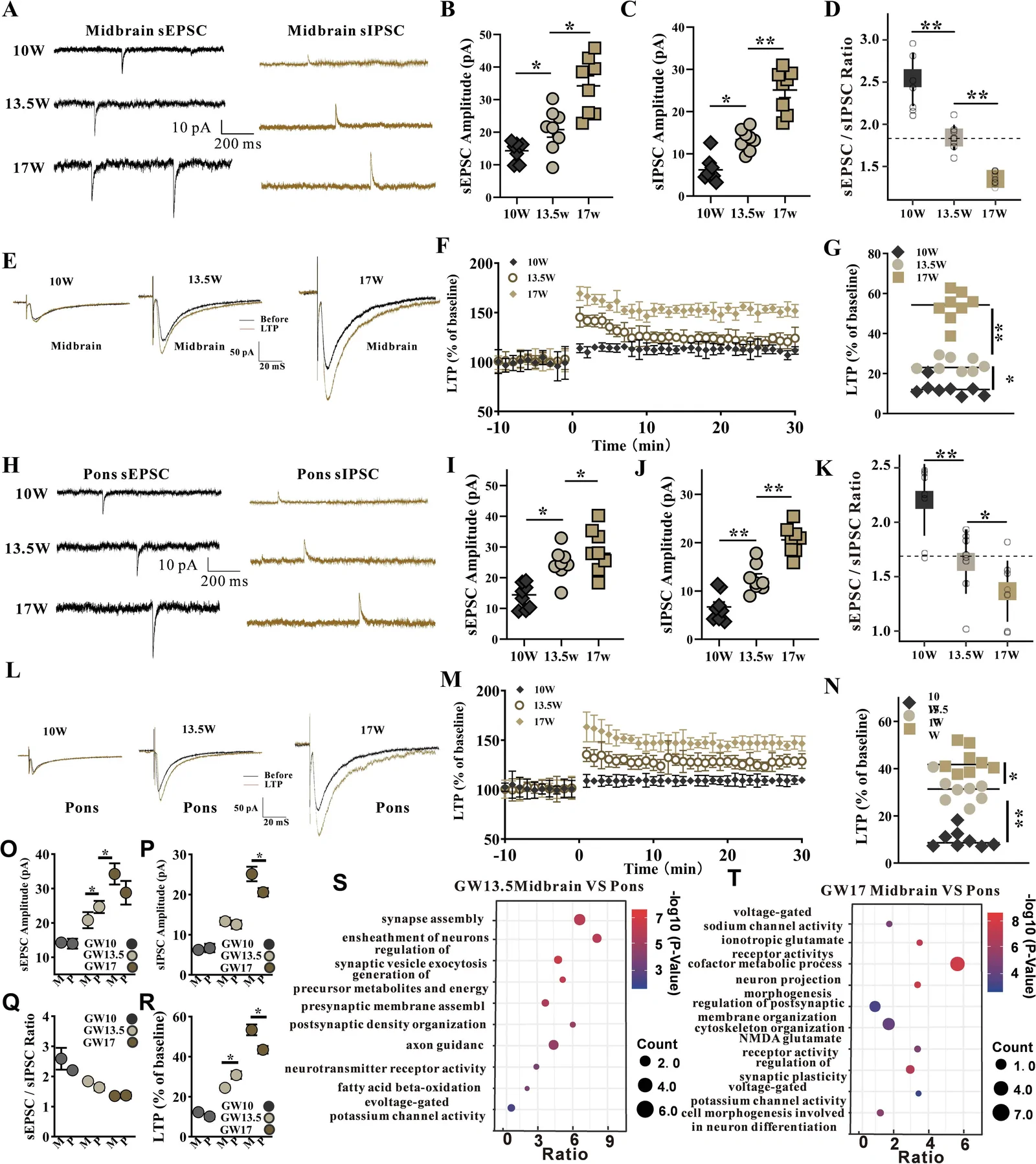
**Spontaneous synaptic activity and LTP in midbrain and pons neurons** (A) Schematic of sEPSCs/sIPSCs in midbrain neurons. (B) sEPSC amplitude in midbrain neurons. (C) sIPSC amplitude in midbrain neurons. (D) sEPSC/sIPSC ratio in midbrain neurons. (E) Schematic diagram of midbrain neuron EPSC-LTP. (F) The amplitude of EPSC percentage changes over 40 min. (G) EPSC-LTP percentage in midbrain neurons. (H) Schematic of sEPSCs/sIPSCs in pons neurons. (I) sEPSC amplitude in pons neurons. (J) sIPSC amplitude in pons neurons. (K) sEPSC/sIPSC ratio in pons neurons. (L) Schematic diagram of pons neuron EPSC-LTP. (M) The amplitude of EPSC percentage changes over 40 min. (N) EPSC-LTP percentage in pons neurons. (O) Comparison of sEPSCs in glutamatergic neurons across developmental time points. (P) Comparison of sIPSCs in glutamatergic neurons across developmental time points. (Q) Comparison of sEPSCs/sIPSCs in glutamatergic neurons across developmental time points. (R) Comparison of sEPSCs/sIPSCs in glutamatergic neurons across developmental time points. (S) Enrichment analysis of GO terms in midbrain and pons glutamatergic neurons at GW13.5. (T) Enrichment analysis of GO terms in midbrain and pons glutamatergic neurons at GW17. Date from 8 neurons per region. Data are presented as mean ± SEM. *p < 0.01, **p < 0.05.

To further explore the developmental patterns of synaptic network, this study recorded sEPSCs and sIPSCs amplitudes and their current ratios in the midbrain and pons glutamatergic neurons at GW10, GW13.5, and GW17. Compared with midbrain glutamatergic neurons at GW10, sEPSC amplitudes increased significantly at GW13.5 and GW17 (Fig. 4B), reflecting progressive maturation of postsynaptic structures. This observation aligns with Kostović’s immunohistochemical evidence indicating a large-scale formation window of excitatory synapses from GW12 to GW20 [48]. Simultaneously, sIPSC amplitudes also increased significantly (Fig. 4C), indicating concurrent enhancement of inhibitory synaptic activity. Further analysis of the excitatory/inhibitory (E/I) ratio revealed that, compared with GW10 neurons, midbrain neurons at GW13.5 and GW17 displayed a significant reduction in E/I ratio (Fig. 4D), indicating strengthened inhibitory regulation that contributes to neural network stabilization. This trend contrasts with reports from primate models, where the E/I ratio in the monkey embryo cortex (equivalent to human GW20-28) remains above 2.0. Single-cell sequencing data from the human fetal cortex (GW13–24) indicate that GABAergic interneurons start expressing mature markers (e.g., KCC2, GAD67) by GW16, earlier than in monkeys, where expression occurs postnatally [49]. This difference suggests that the human midbrain neural network possess an early inhibitory regulatory advantage, potentially better adapted to meet functional requirements for survival. These findings indicate that the midbrain neural network transitions from a rudimentary connectivity state to a more refined regulatory architecture. Additionally, EPSC – LTP in the midbrain was evaluated, revealing a significant increase in LTP amplitude from 24.27 % at GW13.5 to 53.41 % at GW17 (Fig. 4F/G). This period coincides with the GABAergic signaling transition (16–18 weeks) reported by the Tyzio group [50], during which GABA shifts from excitatory to inhibitory, potentially facilitating LTP maturation [51]. Synaptic plasticity increases markedly with fetal age, particularly between GW13.5 and GW17, during which synaptic network development shifts from establishing basic connectivity to refining higher-order functions, thus laying the foundation for complex behavioral and cognitive capacities. In the pons, trends in sEPSCs and sIPSCs amplitudes mirrored those in the glutamatergic neurons, suggesting that the midbrain and pons, as core motor brain regions, share similar developmental regulatory patterns during motor network construction, possibly involving the same molecular pathways. At GW13.5, pons neurons displayed higher sEPSC amplitudes and greater LTP compared with midbrain neurons; by GW17, this pattern reversed, with midbrain neurons exhibiting higher sEPSC amplitudes and greater LTP than pons neurons (Fig. 4O–R). These findings indicate that from GW10 to GW13.5, synaptic network function in the pons is more advanced, whereas between GW13.5 and GW17, the midbrain undergoes accelerated synaptic development. GO term analysis further supported these observations: at GW13.5, pathways related to synapse formation and metabolism in midbrain neurons—including synapse assembly, neurotransmitter receptor activity, and energy metabolism—were relatively underactive. By GW17, pathways related to functional maturation and synaptic transmission were significantly upregulated in the midbrain, whereas structural and morphological pathways showed modest downregulation (Fig. 4S-T). In contrast, pons neurons exhibited pronounced increases in membrane capacitance, indicative of preferential development of structural projections, particularly descending motor fibers. Collectively, these findings suggest that after GW13.5, the midbrain primarily accelerates synaptic transmission and plasticity, whereas the pons preferentially develops structural and projection components. This observation challenges Sarnat’s histological grading system [52], which proposes that the midbrain matures 2–3 weeks earlier than the pons. In summary, our study demonstrates a coordinated enhancement of excitatory and inhibitory synaptic activity, accompanied by a temporally regulated pattern of synaptic plasticity, in the midbrain–pons complex during the mid-gestational period (GW10–GW17). These processes establish the foundation for the emergence of motor and cognitive functions. Furthermore, our results indicate that during mid-gestation, synaptic maturation in midbrain and pons neurons follows region-specific, non-linear developmental trajectories.

**4. Functional defects in midbrain neurons of down syndrome (DS) fetuses at 17 weeks of gestation and exploration of early diagnostic biomarkers**

**Fig. 5:**
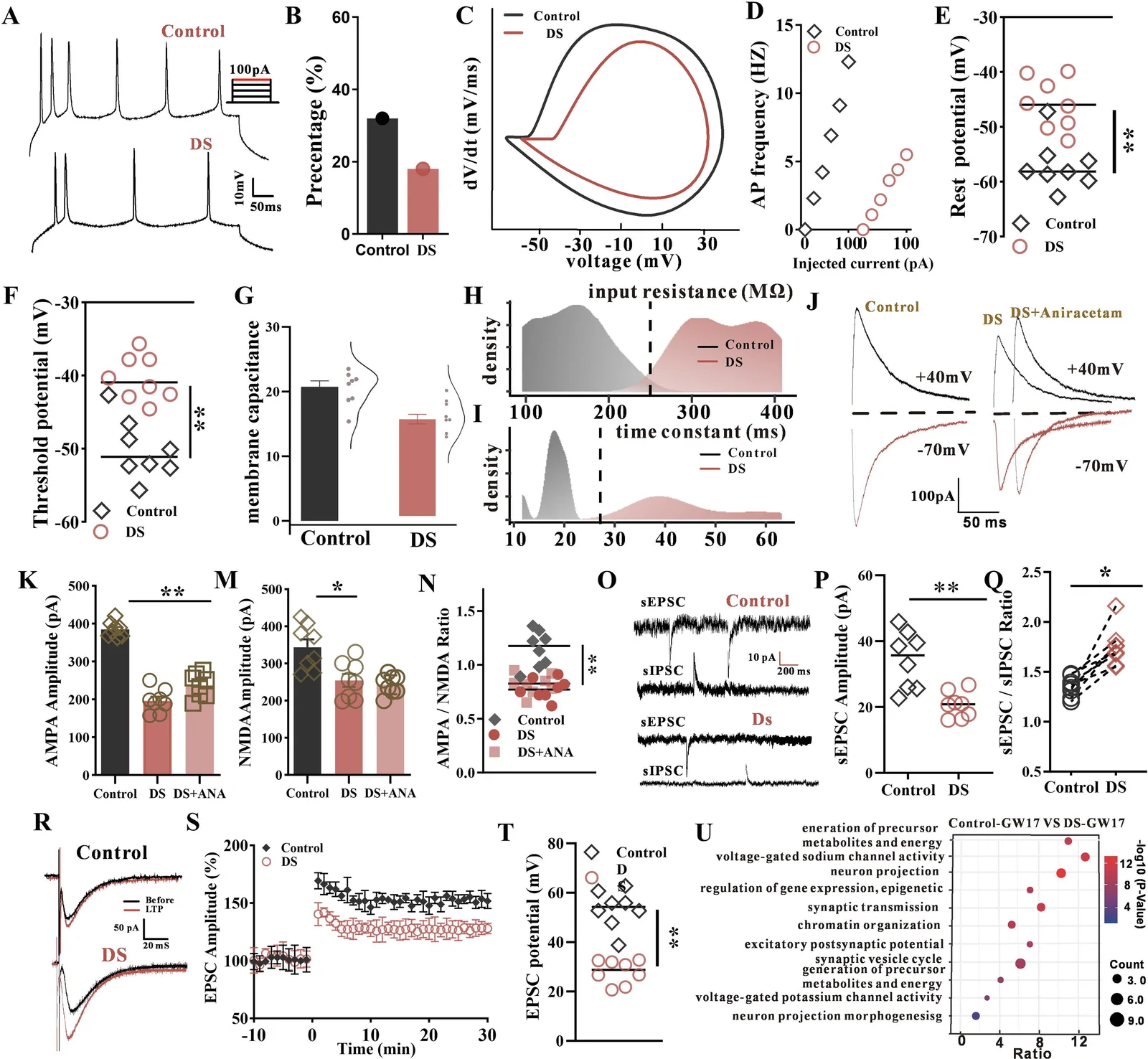
**Electrophysiological and synaptic deficits in GW17 down syndrome (DS) neurons** (A) Schematic of neuronal AP in GW17 Control and GW17 DS group. (B) Ratio of neurons that induced action potentials in healthy Control and DS group. (C) First derivative of neuronal Action potential. (D) AP firing frequency of neurons versus injected current. (E) Resting membrane potential of neuronal AP. (F) Threshold potential of neuronal Action potentials (G) Neuronal membrane capacitance. (H) Input resistance of neurons between GW17 Control group and GW17 DS group. (I) Time constant of neurons between GW17 Control group and GW17 DS group. (J) Schematic representation of AMPAR- and NMDAR-mediated EPSCs in GW17 control, DS, and aniracetam intervention groups. (K) AMPA-EPSCs amplitudes of neurons from the GW17 control, DS, and aniracetam intervention groups. (M) NMADR-EPSCs amplitudes of neurons from the GW17 control, DS, and aniracetam intervention groups. (N) AMPAR/NMDAR - mediated EPSCs ratio. (O) Schematic of SEPSCs/sIPSC currents in neurons of GW17 Control group and GW17 DS group. (P) sEPSC amplitude in neurons of GW17 Control group and GW17 DS group. (Q) Ratio of SEPSCs / sIPSCs in neurons of GW17 Control group and GW17 DS group. (R) Schematic of EPSC-LTP in neurons of GW17 Control group and GW17 DS group. (S) Change in EPSC-LTP percentage over 40 minutes in neurons of GW17 Control group and GW17 DS group. (T) EPSC-LTP percentage in neurons of GW17 Control group and GW17 DS group. (U) GO term-enriched bubble plots of neurons from the control and DS groups at GW17. Data from 8 neurons per group. Data are presented as mean ± SEM. *p < 0.01, **p < 0.05.

We further compared the functional differences of midbrain glutamatergic neurons between the control group and Down syndrome at GW17. Compared to the Control group, DS neurons at GW17 exhibited significant functional network deficits. The responding probability of neuronal AP in the DS group was significantly reduced (Fig. 5B; Control group: 34.7 % ± 4.8 % vs DS group: 16.2 % ± 3.1 %) [53]. Further analysis revealed that the firing frequency of AP in DS neurons was significantly lower than in Control neurons (Fig. 5D; Control group: 12.3 Hz, DS group: 5.5 ± 0.8 Hz, p < 0.01), indicating significant excitability impairment in DS neurons at early developmental stages. Further analysis revealed that DS neurons exhibited significantly depolarized resting membrane potentials (Fig. 5E; −45.83 ± 1.2 mV vs −57.03 ± 1.5 mV, p < 0.01) and an elevated action potential threshold (Fig. 5F; −40.40 ± 1.1 mV vs −50.10 ± 0.8 mV, p < 0.01). These concurrent alterations indicate that DS neurons require stronger depolarizing stimuli to initiate action potentials, resulting in reduced excitability and diminished neural network activity. We also observed significant changes in passive membrane properties, with Cm significantly lower in DS neurons than in controls (Fig. 5G; Control group: 20.75 ± 1.3 pF, DS group: 15.19 ± 0.9 pF, p < 0.05), suggesting reduced membrane surface area. This phenomenon may result from decreased dendritic complexity, consistent with the pathological feature of simplified neuronal structures in DS fetuses [54]. We also observed significant increases in input resistance and membrane time constant in DS neurons at GW17 (Fig. 5H/I), which impede effective neural information integration. RNA-seq and immunohistochemical analyses of DS fetal brain tissue previously demonstrated reduced expression of the dendritic marker MAP2 in midbrain neurons [55], corroborating our electrophysiological findings of decreased dendritic surface area and increased membrane resistance. Regarding synaptic function, GW17 DS neurons exhibited impaired glutamatergic synapse maturation. Both AMPAR mediated EPSCs (Fig. 5K; 196.22 ± 18.7 pA vs 384.58 ± 25.3 pA, p < 0.01) and NMDAR mediated EPSCs (Fig. 5M; 254.55 ± 15.4 pA vs 343.57 ± 20.1 pA, p < 0.05) were significantly reduced, and the AMPAR/NMDAR mediated EPSCs ratio significantly decreased (Fig. 5N; 0.77 ± 0.05 vs 1.15 ± 0.08, p < 0.01), suggesting defects in the postsynaptic density (PSD) structure. This phenomenon is consistent with observations in DS iPSC models [53], where synaptic abnormalities in the PSD have been observed. In addition, DS midbrain neurons were treated with the AMPA receptor allosteric modulator Following aniracetam treatment, AMPA-mediated currents increased, although the change did not reach statistical significance (Fig. 5M). These findings indicate that DS midbrain neurons at GW17 exhibit early deficits in synaptic transmission efficiency, receptor maturation, and PSD structural integration, impairing neuronal information processing and potentially underlying functional abnormalities in neural circuits. At GW17, midbrain neurons in Down syndrome exhibit early deficits in synaptic network excitability, receptor maturation, and structural integration. We further observed that sEPSCs amplitude in DS fetal midbrain neurons was significantly lower than in healthy controls (Fig. 5P; control group: 34.24 pA vs DS group: 20.68 pA, p < 0.01), indicating impairment in postsynaptic function or synaptic structural integrity. The E/I ratio in DS neurons was significantly higher (Fig. 5Q; control group: 1.35 vs DS group: 1.75, p < 0.05), reflecting a relative dominance of inhibitory synapses, which could reduce neural network plasticity and impair cognitive functions. These data suggest that DS neurons display abnormalities in synaptic integration, likely resulting from a combination of postsynaptic receptor deficits and enhanced GABAergic transmission. To explore synaptic plasticity further, we recorded the LTP amplitude of EPSCs. The LTP increase in the DS group was significantly lower than in the control group (Fig. 5S/T; control group: 53.41 % vs DS group: 28.01 %, p < 0.01). This indicates that synaptic plasticity in midbrain neurons of Down syndrome (DS) is impaired, leading to decreased adaptability of the synaptic network. GO term analysis revealed that functional impairments in DS neurons primarily affect ion channel and membrane excitability regulation, synaptic signaling and vesicle cycling, energy metabolism and mitochondrial function, and chromatin organization and epigenetic regulation (Fig. 5U). These molecular and cellular abnormalities result in decreased membrane capacitance, impaired dendritic spine development, reduced glutamatergic transmission efficiency, and disrupted transcriptional timing. Collectively, these findings demonstrate that by GW17, DS midbrain neurons exhibit early deficits in synaptic network function, encompassing excitability, receptor maturation, synaptic transmission efficiency, and plasticity. These impairments are likely to compromise normal maturation of motor network function. Our findings highlight potential targets for physiology-based, non-invasive prenatal diagnostic biomarkers and identify GW13.5–GW17 as a critical developmental window for interventions to mitigate neurodevelopmental delay in DS.

## Discussion

This study, through systematic electrophysiological analysis, is the first to reveal the functional development of early neural networks in the midbrain- pons region (GW10-GW17) in human fetuses and to identify electrophysiological abnormalities specific to Down syndrome (DS) fetuses. These findings fill the gap in understanding the functional development of synaptic during human embryonic development and provide a new theoretical basis for the early diagnosis and intervention of neurodevelopmental disorders (NDDs).

In healthy fetuses, the development of the midbrain- pons region exhibits clear spatiotemporal dynamics: action potential frequency significantly increases from GW10 to GW17, tightly coupled with the window of spontaneous limb movements (GW13-GW15). This process is accompanied by changes in membrane capacitance—rapid dendritic growth occurs from GW10 to GW13.5, while the growth rate slows after GW13.5. Notably, compared to the midbrain, continuous membrane capacitance increase in the pons from GW13.5 to GW17 aligns with the motor conduction pathway development observed in primate studies, possibly explaining the embryonic origin of human motor coordination. The trajectory of synaptic function development further reveals the hierarchical regulation of neural network maturation. During GW10-GW13.5, AMPAR receptor-mediated excitatory postsynaptic currents (AMPAR-EPSC) rapidly increase in the midbrain and pons, laying the foundation for fast neural signal transmission to meet early motor control demands. From GW13.5 to GW17, NMDA receptor-mediated currents (NMDAR-EPSC) significantly increase, accompanied by a decrease in the AMPAR/NMDAR −EPSCs ratio, reflecting enhanced NMDA receptor function that fine-tunes synaptic plasticity, marking the transition to more complex neural information processing From GW13.5 to GW17, the AMPAR/NMDAR current ratio in the midbrain rises from 0.50 to 1.14, reflecting the staged maturation of glutamatergic synapses. Synaptic enhancement in the midbrain and pons occurs earlier than in the macaque prefrontal cortex [56], suggesting that synaptic maturation in the motor system may precede that in higher-order cognitive regions. This indirectly supports the hypothesis of layered development of motor and cognitive functions, where motor circuits mature early in the fetus, laying the foundation for subsequent cognitive functions. Notably, this increase sharply rises from GW13.5 to GW17, synchronized with a significant enhancement of EPSC-LTP amplitude, suggesting that AMPA receptor insertion into the postsynaptic membrane may be the core mechanism for opening the synaptic plasticity window. In contrast, NMDAR-EPSCs in the pons neurons significantly increases from GW10 to GW13.5, indicating earlier cross-modal integration in this region. This feature is likely associated with the pons’ relay function in sensory-motor pathways, supporting its role in theta-gamma cross-frequency coupling—a mechanism critical for real-time motor coordination in the adult pons—and further reinforcing the pons’ early central role in motor control and sensory-motor integration [[57], [58]]. The synchronous growth of sEPSCs / sIPSCs amplitude indicates “competitive synergy” between excitatory and inhibitory systems. The rapid maturation of excitatory synapses drives neural networks, while the higher growth rate of inhibitory synapses (sIPSCs increase 2.4 times that of sEPSCs) reduces the E/I ratio from 2.5 to 1.2, stabilizing the network. Compared to macaque embryos (E/I > 2.0 until GW28), human fetuses reach E/I = 1.2 at GW17, possibly due to premature differentiation of GABAergic interneurons. In GW17 DS fetuses, we observed a typical set of electrophysiological abnormalities, including a significant reduction in action potential frequency, a decrease in the AMPAR/NMDAR-EPSCs ratio, and a significant increase in the E/I ratio These abnormalities indicate an early imbalance in the excitatory-inhibitory homeostasis of the neural network at GW17. The synergistic effects of depolarization of the resting membrane potential and increased input resistance may reduce the stability of action potential firing, disrupting the balance from the midbrain substantia nigra to the basal ganglia, ultimately affecting normal neural circuit function, closely aligning with clinical observations of motor retardation in DS patients. The significant attenuation of AMPAR −EPSCs and the associated long-term potentiation (LTP) induction defect suggest that synaptic plasticity dysfunction may be an early electrophysiological marker of DS. The observation of LTP defects in GW17 DS fetuses suggests that intrauterine intervention should occur before the17th week of pregnancy, offering a new theoretical framework for the timing of fetal medical interventions. The midbrain- pons functional development map constructed in this study fills a critical gap in the functional development of human embryos and reveals the close connection between ion channel expression, synaptic receptor dynamics, and neural network plasticity. After GW13.5, the midbrain preferentially develops synaptic networks, while the pons preferentially undergoes morphological and fiber expansion. This provides new guidelines for cultivating stem cell-derived midbrain organoids. The critical window from GW13.5 to GW17 unveils the potential for maternal drug delivery strategies, such as potassium channel regulators or AMPA allosteric modulators, to help improve neural network function. Future research should validate the association between these electrophysiological markers and motor and cognitive development in children through longitudinal cohorts and explore the potential application of transcranial magnetic stimulation (TMS) for non-invasive regulation of fetal neural circuits. Although this study is the first to chart the electrophysiological development map of human fetal motor circuits, further efforts should deepen the understanding of individual developmental differences and their relationship with genetic variations by expanding the sample size, integrating multi-center data, and developing high spatiotemporal resolution imaging techniques, ultimately advancing precision medicine in prenatal neurodevelopmental disease diagnosis and intervention.

## Funding

This study was supported in part by the Basic research project of Science and Technology Innovation Action Plan Innovation (23JC1401800/23JC1401801/23JC1401802), the Natural Science Foundation of Shanghai (20ZR1448400).

## Author Contributions

Yongkang Wu^1^, Yi Dong*, and Qizhi He* conceived and designed the study. Yongkang Wu^1^, Hao Peng^1^, Yanni Chen^1^ performed the experiments and collected data. Fanglin Wang, Jian Wang, Jinli Ou, Yan Cui, and Yuhua Zheng, Yigang Dong, and Qingmiao Hu contributed to data analysis and interpretation. Jianqiang Huang*, Yi Dong*, and Qizhi He* supervised the project and acquired funding. Yongkang Wu and Yingmei Fu drafted the manuscript, Yi Dong* and Qizhi He* provided critical revision. All authors read and approved the final manuscript.

## Ethical statement

Human fetal tissue samples were obtained from patients undergoing elective pregnancy termination at the Pingdingshan Maternity and Infant Health Hospital. The collection and use of human fetal tissue were conducted in accordance with the legal and ethical principles outlined in the Declaration of Helsinki and were approved by the Reproductive Research Ethics Committee of Pingdingshan Maternity and Infant Health Hospital. All donors provided informed consent prior to tissue collection. All procedures were conducted in compliance with the “Interim Measures for the Administration of Human Genetic Resources” issued by the Ministry of Science and Technology of China.

## Declaration of competing interest

The authors declare that they have no known competing financial interests or personal relationships that could have appeared to influence the work reported in this paper.

## Appendix A Supplementary data

Supplementary Data 1

Supplementary Data 2 (A) GW10 sample. (B) GW13.5 sample. (C) GW17 control sample. (D) GW17 DS sample. (E) Chromosomal copy number analysis of the DS sample genome. (F) Chromosome 21 copy number in DS samples.
